# Efficacy and safety of growth hormone treatment in children with short stature: the Italian cohort of the GeNeSIS clinical study

**DOI:** 10.1007/s40618-015-0418-0

**Published:** 2015-12-28

**Authors:** M. Cappa, L. Iughetti, S. Loche, M. Maghnie, A. Vottero, GeNeSIS National Board on behalf of the GeNeSIS Italian Investigators, Franco Antoniazzi, Luciano Beccaria, Sergio Bernasconi, Domenico Caggiano, Manuela Caruso-Nicoletti, Alessandra Catucci, Francesco Chiarelli, Stefano Cianfarani, Anna Rita Colucci, Francesca De Rienzo, Raffaele Di Pumpo, Alessandra Di Stasio, Giovanni Farello, Leonardo Felici, Pasquale Femiano, Luigi Garagantini, Claudia Giavoli, Nella Augusta Greggio, Laura Guazzarotti, Daniela Larizza, Maria Rosaria Licenziati, Antonella Lonero, Maria Cristina Maggio, Alberto Marsciani, Patrizia Matarazzo, Laura Mazzanti, Beatrice Messini, Flavia Napoli, Anna Maria Pasquino, Laura Perrone, Sabrina Pilia, Alba Pilotta, Marzia Piran, Gabriella Pozzobon, Barbara Predieri, Michele Sacco, Mariacarolina Salerno, Antonina Tirendi, Graziamaria Ubertini, Silvia Vannelli, Malgorzata Wasniewska, Maria Zampolli, Martina Zanotti, Gianvincenzo Zuccotti

**Affiliations:** Endocrinology and Diabetes Unit, Bambino Gesù Children’s Hospital, Rome, Italy; Pediatric Unit, University of Modena and Reggio, Modena, Italy; Pediatric Endocrinology, Ospedale Microcitemico ASL Cagliari, Cagliari, Italy; Department of Pediatrics, IRCCS Giannina Gaslini, University of Genova, Genoa, Italy; Medical Diabetes Group, Eli Lilly and Company, 50019 Sesto Fiorentino, Italy

**Keywords:** Pediatric GH treatment, Short stature, Growth, Safety, Final height

## Abstract

**Purpose:**

We examined auxological changes in growth hormone (GH)-treated children in Italy using data from the Italian cohort of the multinational observational Genetics and Neuroendocrinology of Short Stature International Study (GeNeSIS) of pediatric patients requiring GH treatment.

**Methods:**

We studied 711 children (median baseline age 9.6 years). Diagnosis associated with short stature was as determined by the investigator. Height standard deviation score (SDS) was evaluated yearly until final or near-final height (*n* = 78). Adverse events were assessed in all GH-treated patients.

**Results:**

The diagnosis resulting in GH treatment was GH deficiency (GHD) in 85.5 % of patients, followed by Turner syndrome (TS 6.6 %). Median starting GH dose was higher in patients with TS (0.30 mg/kg/week) than patients with GHD (0.23 mg/kg/week). Median (interquartile range) GH treatment duration was 2.6 (0.6–3.7) years. Mean (95 % confidence interval) final height SDS gain was 2.00 (1.27–2.73) for patients with organic GHD (*n* = 18) and 1.19 (0.97–1.40) for patients with idiopathic GHD (*n* = 41), but lower for patients with TS, 0.37 (−0.03 to 0.77, *n* = 13). Final height SDS was >−2 for 94 % of organic GHD, 88 % of idiopathic GHD and 62 % of TS patients. Mean age at GH start was lower for organic GHD patients, and treatment duration was longer than for other groups, resulting in greater mean final height gain. GH-related adverse events occurred mainly in patients diagnosed with idiopathic GHD.

**Conclusions:**

Data from the Italian cohort of GeNeSIS showed auxological changes and safety of GH therapy consistent with results from international surveillance databases.

## Introduction

Short stature in children is an important problem that should be diagnosed and managed appropriately in order to promote normal height [1]. Human recombinant growth hormone (GH) therapy was introduced in 1985 for the indication of GH deficiency (GHD), and since then, thousands of children with short stature have achieved improved height outcomes. GHD remains the main indication for GH therapy in children and can be due to varied etiology, with both congenital and acquired causes [2, 3], although for the majority the cause remains unknown and is termed idiopathic GHD. GH therapy has subsequently been approved in the USA and Europe for other pediatric conditions that result in short stature, including Turner syndrome, being born small for gestational age (SGA) with failure to attain normal growth, Prader-Willi syndrome, chronic renal insufficiency, short stature homeobox-containing gene (SHOX) deficiency, and, in the USA but not Europe, Noonan syndrome and idiopathic short stature [1, 4–11].

GH therapy in pediatric patients is generally considered safe, with serious adverse events reported rarely, particularly with currently approved doses [12–15]. Certain specific conditions such as benign intracranial hypertension, scoliosis and slipped capital femoral epiphysis have been observed shortly after starting GH in small numbers of treated patients [16]. Concerns have also been raised about the possibility of long-term alterations in glucose metabolism and an association with the occurrence of neoplasms, but there is little evidence for either except in patients with preexisting risk factors [13, 17–22].

Continued surveillance studies on efficacy and safety of GH therapy in children remain important, and long-term outcomes and safety have been documented in large international databases of GH-treated patients. These include the Genetics and Neuroendocrinology of Short Stature International Study (GeNeSIS) sponsored by Eli Lilly and Company, the Kabi International Growth Study (KIGS) sponsored by Pfizer, the National Cooperative Growth Study (NCGS) sponsored by Genentech and the NordiNet^®^ International Outcome Study (IOS) sponsored by Novo Nordisk. These multinational studies provide global information, giving an overview of the impact of GH treatment in children. However, there may be ethnic or regulatory differences across and between countries that affect treatment uptake. These differences may involve funding, indications and patient ages required for treatment initiation, and factors affecting treatment outcomes, such as patient attributes that can positively or negatively affect the efficacy and safety of treatment. It is therefore important to identify country-specific data on the effects of GH therapy, which have currently been reported to a very limited extent. The objective of the current study was to evaluate the auxological changes occurring during GH treatment in a population of Italian pediatric patients, based on data collected in the observational GeNeSIS program. As well as the Italian cohort overall, we have examined data within specific diagnostic groups where patient numbers allow.

## Methods

### Patient population

GeNeSIS is a prospective, multinational, open-label, post-marketing surveillance program designed to examine the long-term safety and efficacy of GH (Humatrope^®^, Eli Lilly and Company, Indianapolis, USA) administered for short stature in children; the study also allows certain GH-untreated cases to be followed. The multinational study is performed according to the ethical principles of the Declaration of Helsinki and is approved by all appropriate local ethics review committees, with written informed consent for data collection, processing and publication provided by the parents or a legal guardian for each child according to national laws and regulations.

Patients enrolled in GeNeSIS were either starting GH treatment or already being treated with GH for the improvement of growth and did not present with closed epiphyses, although patients who had epiphyseal closure during participation could remain in the study. GeNeSIS is an observational study, and therefore, all diagnoses were as reported by the attending physician, and treatment decisions were completely at the discretion of the participating investigator. However, diagnoses and treatments were to be performed according to standard pediatric endocrinology practice, with GHD defined using current guidelines [23]. A total of 60 study centers in Italy enrolled patients into GeNeSIS, and the present study examined data from the start of the study in 1999 until data lock in September 2012.

### Study evaluations

On entry to the study, baseline values for each patient were recorded for chronological age, bone age, height, height velocity (HV), weight, and genetic target height from the sex-adjusted average of parental heights. Medical history, including diagnosis of the cause of short stature and prior medications, was recorded. Pubertal stage was evaluated according to the Tanner classification. Changes in height, HV and pubertal stage were determined in all study visits during follow-up, which occurred at least once yearly. For patients who reached final height during the study, final height and difference from genetic target height were also evaluated.

GeNeSIS is a multinational study, and therefore, the same reference data were applied across participating countries for calculation of standard deviation score (SDS). Thus, height SDS was determined according to the 2000 US National Center for Health Statistics standards [24], and HV SDS was calculated using prespecified age- and gender-matched reference data [25].

Safety analysis was based on adverse events in all Italian patients receiving GH therapy; relationship to GH treatment and severity of each event were as determined by the investigators. Serious adverse events were classified as those that resulted in death, hospitalization, persistent or significant disability, or congenital abnormality in offspring of the patient, or were considered life-threatening or significant in the investigator’s opinion. Treatment-emergent adverse events were defined as events that first occurred or worsened in severity after initiation of GH therapy and, thus, were evaluated only in patients who had at least one post-baseline follow-up visit. Special attention was paid to reports of neoplastic disease and alterations in glucose metabolism. All adverse events were categorized according to the Medical Dictionary for Regulatory Authorities (MedDRA), version 11.0.

### Statistics

Diagnosis was evaluated for all children with sufficient information. Patients who were GH naïve at entry and had baseline and 1-year height SDS data were evaluated by diagnostic groups for changes in auxological parameters at 1 year. A subgroup, comprising patients who were GH naïve and had yearly height measurements for 4 years, was evaluated for auxological changes overall and by diagnostic category where patient numbers were sufficient. A further subgroup of patients who were either GH naïve or already GH-treated at entry was assessed for auxological changes at final or near-final height, defined as having reached one of the following criteria: closed epiphyses, height velocity <2 cm/year or bone age >14 years for girls or >16 years for boys. Data are presented as medians with first and third quartiles (Q1–Q3) for variables that may have a skewed distribution (baseline age, GH dose, time in study), as mean with 95 % confidence interval (CI) for efficacy-related continuous variables, and as frequency and percentage for categorical variables. Outcomes were primarily assessed from examination of the overlap of means and 95 % CI. Statistical analyses were conducted using SAS^®^ 9.1.

## Results

### Patient disposition and characteristics

**Fig. 1:**
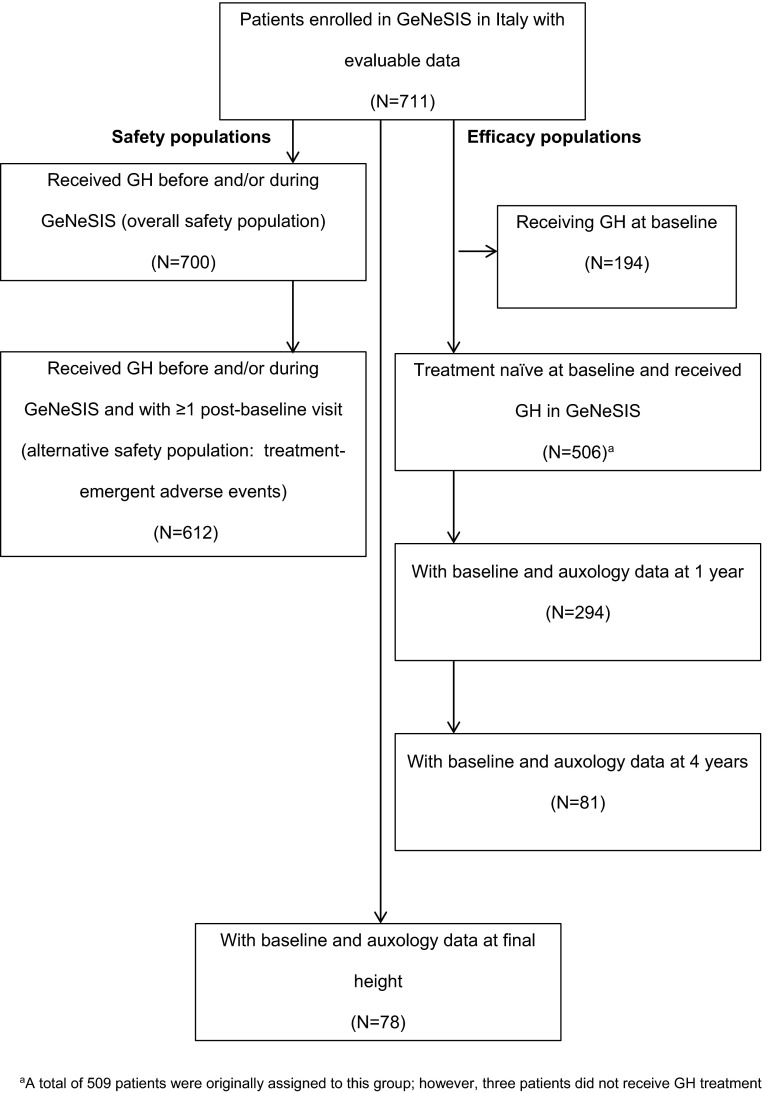
Flow chart showing numbers of patients in Italy available for specific analyses

At the time of analysis, there were 711 children (58.9 % male, 41.0 % female) with evaluable data enrolled in GeNeSIS in Italy; the flow of patients through this analysis is shown in Fig. 1. The majority were Caucasian (90.4 %), with ethnic origin unspecified for 6.6 %. At GeNeSIS entry, 27.3 % were already receiving GH and 71.6 % were GH naïve; 11 patients (1.5 %) did not receive GH treatment at any time during the study. The diagnoses associated with short stature leading to initiation of GH treatment are summarized in Table 1 for 662 patients with sufficient evaluable information. The most frequent diagnosis was GHD (85.5 % of GH-treated total), which was reported to be idiopathic in 73.5 % of GHD cases and due to organic causes in 26.1 % of GHD cases (Table 1). The second largest diagnostic category resulting in GH treatment was SHOX deficiency disorders (7.6 %), most commonly Turner syndrome (6.6 %).

**Table 1:** Primary diagnosis and secondary level diagnoses in 662 children in Italy treated with GH for short stature

Primary diagnosis and secondary diagnosis	*n* (% of total)	% of primary diagnosis
GH deficiency	566 (85.5)	
Idiopathic	416 (62.8)	73.5
Classic	383 (57.9)	67.7
Neurosecretory dysfunction	16 (2.4)	2.8
Organic	148 (22.4)	26.1
Congenital	123 (18.6)	21.7
Abnormal pituitary development^a^	109 (16.5)	19.3
Clinical syndromes^b^	4 (0.6)	0.7
Genetic defect	4 (0.6)	0.7
Other CNS malformations	2 (0.3)	0.4
Acquired	25 (3.8)	4.4
Intracranial tumor^c^	20 (3.0)	3.5
Cranial irradiation	3 (0.5)	0.5
Histiocytosis	1 (0.2)	0.2
Other	1 (0.2)	0.2
SHOX deficiency syndromes	50 (7.6)	
Turner syndrome	44 (6.6)	88.0
Léri-Weill syndrome	5 (0.8)	10.0
Other diagnosis	1 (0.2)	2.0
Other causes of short stature or reduced linear growth	23 (3.5)	
Genetic defect	6 (0.9)	26.1
Other^d^	17 (2.6)	73.9
Small for gestational age	20 (3.0)	
Idiopathic short stature	2 (0.3)	
Other defects of GH axis (bioinactive GH)	1 (0.2)	

Information was as provided by the investigator and was not always provided at lower levels of diagnosis; investigator-provided diagnoses were assigned to a predefined hierarchical diagnostic tree to classify the primary cause of short stature and establish appropriate diagnostic groups

^a^Pituitary hypoplasia (55), ectopic posterior pituitary (31), pituitary aplasia (7), pituitary stalk defect (6), septo-optic dysplasia (8), other (1)

^b^Midline palatial defect (4)

^c^Craniopharyngioma (13), medulloblastoma (2), germinoma (2), glioma (1), ependymoma (1), pituitary adenoma (1)

^d^Noonan syndrome (3), chronic renal failure (2), inflammatory bowel disease (1), other (11)

Among all patients, 21.0 % were aged ≤5 years at the time of diagnosis, 35.3 % were aged >5 to ≤10 years, and 40.6 % were aged >10 to ≤15 years; a slightly higher proportion of females than males were in the range >5 to ≤10 years (females 38.2 %, males 33.6 %), whereas a lower proportion of females were in the range >10 to ≤15 years (38.2 %, 42.1 %). At entry to GeNeSIS, Tanner stage 1 was reported for the majority of both female (81.3 %) and male (81.1 %) patients. At the last visit before data lock, 40.5 % of the male and 40.6 % of the female children were still prepubertal (Tanner stage 1), whereas pubertal stage ≥3 was reported for 46.3 % of male and 44.8 % of female patients.

For the GH-treated patients overall, median (Q1–Q3) age at start of GH treatment was 9.6 (5.8–11.9) years. Median (Q1–Q3) starting GH dose for patients overall was 0.23 (0.19–0.26) mg/kg/week; starting dose was higher for patients with Turner syndrome, 0.30 (0.26–0.34) mg/kg/week, and for the 2 patients with chronic renal insufficiency, 0.31 (0.28–0.34) mg/kg/week, but similar across the other diagnostic categories. Median (Q1–Q3) follow-up in GeNeSIS was 2.0 (0.6–3.7) years, with follow-up ≥4 years for 151 patients; because 27 % had started GH before study entry, median total time on GH was 2.6 (1.0–4.5) years. Discontinuation of GH therapy was reported for 152 patients; the reported reason was physician decision for 24, patient/parent decision for 24 and lack of efficacy for 7 patients. Discontinuation due to adverse events was reported for 2 patients, with death reported for one of these patients.

### Auxological data during GH therapy

Auxological data at 1 year were available for a total of 294 patients (184 males, 110 females) who were treatment naïve at baseline and received GH during the study. Of these, 193 had idiopathic GHD (187 isolated GHD, 6 multiple pituitary hormone deficiencies) and 60 had organic GHD (Table 2). Age at start of GH treatment was lower for the organic GHD group than for the idiopathic GHD group. Height SDS was similar, but deficit from target height was greater for those with organic GHD (Table 2). Responses after 1 year of GH therapy were similar for the two groups, and target height deficit remained greater in the organic GHD group. Patients with Turner syndrome had baseline characteristics similar to both GHD groups. However, although the administered GH dose was greater, the response at 1 year in Turner syndrome patients was less than for GH-deficient patients. Short stature patients born SGA and patients with SHOX deficiency other than Turner syndrome showed similar characteristics at baseline and a similar response at 1 year to patients with idiopathic GHD (Table 2).

**Table 2:** Patient characteristics and auxological data at baseline and after 1 year of GH treatment, for patients who were GH treatment naïve at study entry and had at least 1 year of follow-up, by primary diagnostic category

	Idiopathic GHD (*n* = 193)^a^	Organic GHD (*n* = 60)^b^	Turner syndrome (*n* = 16)	SHOX deficiency (*n* = 5)^c^	SGA (*n* = 8)^d^
Baseline					
Age (years)	9.8 (9.3–10.3)	7.9 (6.7–9.1)	9.0 (7.1–10.8)	8.6 (6.4–10.8)	9.9 (7.4–12.4)
Bone age SDS	−2.18 (−2.38 to −1.99)	−2.02 (−2.49 to −1.54)	−1.54 (−2.40 to −0.68)	NA	−1.18 (−2.45 to 0.09)
Height velocity (cm/year)	4.83 (4.12 to 5.55)	5.37 (4.32 to 6.43)	3.50 (2.18 to 4.83)	NA	3.99 (1.97 to 6.01)
Height velocity SDS	−1.24 (−1.57 to −0.91)	−1.05 (−1.61 to −0.49)	−2.75 (−4.39 to −1.11)	NA	−1.28 (−2.22 to −0.34)
Height SDS	−2.40 (−2.51 to −2.30)	−2.55 (−2.86 to −2.24)	−2.30 (−2.59 to −2.01)	−2.74 (−3.77 to −1.71)	−2.91 (−3.45 to −2.38)
Target height SDS deficit^e^	−1.40 (−1.53 to −1.27)	−2.14 (−2.53 to −1.75)	−2.01 (−2.40 to −1.63)	−1.37 (−1.84 to −0.89)	−1.49 (−2.23 to −0.74)
GH dose (mg/kg/week)	0.23 (0.22–0.24)	0.23 (0.21–0.25)	0.31 (0.27–0.35)	0.23 (0.21–0.26)	0.23 (0.19–0.27)
Stimulated peak GH (µg/l)	6.43 (5.38–7.47)	6.25 (4.59–7.91)	NA	NA	15.14 (10.96–19.33)
Year 1					
Height velocity (cm/year)	8.84 (8.51–9.17)	9.25 (8.49–10.00)	7.43 (6.24–8.62)	7.25 (5.30–9.21)	8.17 (7.12–9.21)
Height velocity SDS	2.30 (2.04–2.57)	2.42 (1.83–3.02)	1.40 (0.67–2.12)	1.98 (−0.13 to 4.09)	1.63 (0.92–2.35)
Height SDS	−1.89 (−2.00 to −1.77)	−1.86 (−2.11 to −1.61)	−1.96 (−2.30 to −1.61)	−2.27 (−3.49 to −1.06)	−2.52 (−3.18 to −1.87)
Height SDS gain	0.55 (0.50–0.60)	0.70 (0.52–0.88)	0.32 (0.15–0.48)	0.46 (0.18–0.74)	0.41 (0.29–0.53)
Target height SDS deficit^e^	−0.88 (−1.03 to −0.74)	−1.44 (−1.78 to −1.09)	−1.67 (−2.16 to −1.19)	−0.90 (−1.49 to −0.31)	−1.08 (−1.88 to −0.29)

Data show mean (95 % CI); patient numbers are for those with height SDS at baseline and 1 year, but not all patients had all other information

*NA* no available data, *SDS* standard deviation score, *SGA* short for gestational age

^a^68.4 % male

^b^61.7 % male

^c^40.0 % male

^d^50.0 % male

^e^Height SDS minus target height SDS

**Fig. 2:**
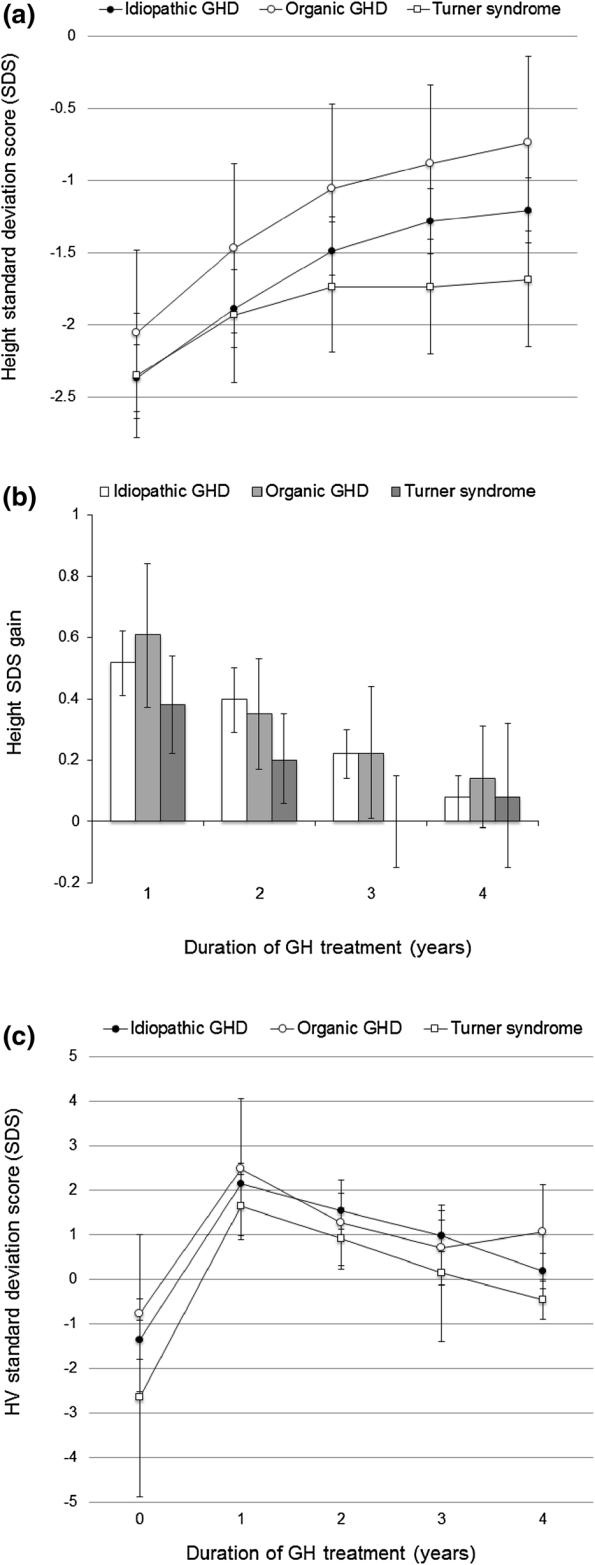
Change in height SDS **a**, gain in height SDS from baseline **b**, and change in height velocity **c**, by duration of GH treatment, for patients with idiopathic GH deficiency (GHD, *n* = 54), organic GHD (*n* = 15) or Turner syndrome (*n* = 9), who were GH treatment naïve at study entry and had at least 4 years of follow-up

There were 81 patients who were GH naïve at study entry and had data available for at least 4 years of GH treatment. Changes in height SDS and HV SDS during 4 years of GH treatment are shown in Fig. 2 for diagnostic groups that had sufficient evaluable information. For both the idiopathic GHD and organic GHD groups, there was an increase in HV SDS, which reduced over time as expected, but was sustained through 4 years of GH therapy. For patients with a diagnosis of Turner syndrome, there was a mean gain in height SDS, particularly in the first year of treatment, but it was lower than that for patients with GHD.

Auxological information was available for a total of 78 patients (46 boys, 32 girls, GH naïve or already GH-treated at study entry) considered to have reached final (or near-final) height. Data at baseline and at final height are shown in Table 3 for patients with idiopathic GHD, organic GHD and Turner syndrome. The organic GHD patients started GH treatment at a slightly younger mean age and were treated for a longer period than the idiopathic GHD group, although the final GH dose was lower for the organic GHD group. Mean final height SDS gain was higher for the organic GHD group than for the idiopathic GHD group, and both GHD groups had mean final height SDS close to target height SDS. The mean height SDS gain for the Turner syndrome group was lower than that for either of the GHD groups, and final height SDS remained below target height SDS.

**Table 3:** Characteristics and auxological data at baseline and at final (or near-final) height for patients with idiopathic isolated GH deficiency (GHD), organic GHD or Turner syndrome, who were either GH treated or GH naïve at study entry

	Idiopathic GHD (*n* = 41)^a^	Organic GHD (*n* = 18)^b^	Turner syndrome (*n* = 13)
Baseline			
Age	11.1 (9.9–12.4)	9.3 (6.7–11.9)	10.3 (8.7–11.9)
Height SDS	−2.04 (−2.27 to −1.82)	−2.60 (−3.43 to −1.77)	−2.38 (−2.73 to −2.03)
Target height SDS deficit^c^	−1.36 (−1.63 to −1.10)	−1.98 (−2.92 to −1.04)	−1.87 (−2.28 to −1.47)
Body weight (kg)	35.1 (30.5–39.7)	28.4 (19.2–37.7)	30.0 (23.1–37.0)
GH dose (mg/kg/week)	0.23 (0.22–0.25)	0.17 (0.13–0.22)	0.30 (0.25–0.35)
Stimulated peak GH (µg/l)	5.15 (4.19–6.10)	3.23 (1.57–4.88)	NA
Final height			
Age	16.8 (16.3–17.2)	17.7 (16.8–18.6)	16.3 (15.5–17.1)
Height SDS	−0.86 (−1.12 to −0.60)	−0.60 (−1.09 to −0.11)	−2.01 (−2.46 to −1.56)
Height SDS gain	1.19 (0.97–1.40)	2.00 (1.27–2.73)	0.37 (−0.03 to 0.77)
Target height SDS deficit^d^	−0.17 (−0.41 to 0.07)	0.02 (−0.58 to 0.62)	−1.49 (−2.09 to −0.90)
GH duration (years)	5.38 (4.22–6.54)	8.19 (5.89–10.49)	5.10 (2.91–7.30)
Body weight (kg)	59.7 (55.6–63.8)	65.4 (58.1–72.7)	50.3 (45.5–55.1)
Final GH dose (mg/kg/week)	0.21 (0.19–0.23)	0.13 (0.09–0.17)	0.26 (0.23–0.29)
Final height SDS >−2 (%)	88 %	94 %	62 %

Data show mean (95 % confidence intervals), except final height SDS >−2, which shows percentage of patients

*NA* no available data, *SDS* standard deviation score

^a^70.7 % male

^b^77.8 % male

^c^Baseline height SDS minus target height SDS

^d^Final height SDS minus target height SDS

### Safety outcomes

There were 700 Italian children (412 males, 287 females, 1 gender not specified) included in the overall safety analysis. Mean duration of GH treatment was 3.4 years (95 % CI 3.2–3.7 years). One death was reported, which was of a 3.7-year-old boy with idiopathic isolated GHD who had been treated with GH for 0.8 years when he experienced respiratory failure, associated with a respiratory infection, over a period of approximately 3 months; the respiratory failure was considered by the investigator to be unrelated to GH treatment. At least one serious adverse event was reported for 11 (1.6 %) of the 700 patients during the study (Table 4). These were mostly reported among patients diagnosed with GHD, with no serious adverse events reported among the 44 Turner syndrome patients. Of the serious adverse events reported, 2 were classified by the investigators as related to GH treatment: an event of ketotic hypoglycemia in an 8.2-year-old boy with idiopathic isolated GHD treated with GH for 0.7 years, and a recurrence of craniopharyngioma in a 10.2-year-old boy who had multiple pituitary hormone deficiencies, although the diagnosis was not originally specified (hence, the patient was not included in a specific diagnostic group). Craniopharyngioma recurred after treatment with GH for 2.9 years, although an earlier recurrence in the patient was reported prior to starting GH. Adverse events were reported as the reason for discontinuation for two patients. One was the patient who experienced the fatal event of respiratory failure. The second was an event of impaired glucose tolerance, identified from an oral glucose tolerance test, in a 13.1-year-old boy after 2.5 years of GH treatment for idiopathic GHD.

**Table 4:** Serious adverse events and treatment-emergent adverse events reported in all Italian GH-treated patients and in those diagnosed with idiopathic and with organic GH deficiency (GHD) who had at least one post-baseline visit

	Patients reporting adverse events, *n* (% of *N*)		
	All patients (*N* = 612)	Idiopathic GHD (*N* = 358)	Organic GHD (*N* = 135)
Serious adverse events^a^	11 (1.6)	5 (1.2)	4 (2.7)
Serious adverse events considered GH-related^*^	2 (0.3)	1 (0.2)	0
Treatment-emergent adverse events^b^	130 (21.2)	65 (18.2)	42 (31.1)
Headache	12 (2.0)	8 (2.2)	2 (1.5)
Hypothyroidism^c^	7 (1.1)	2 (0.6)	3 (2.2)
Hypogonadism^c^	6 (1.0)	–	4 (3.0)
Secondary adrenal insufficiency	6 (1.0)	–	6 (4.4)
Varicella	5 (0.8)	3 (0.8)	2 (1.5)
Scoliosis	5 (0.8)	1 (0.3)	4 (3.0)
Secondary hypothyroidism	4 (0.7)	–	4 (3.0)
Ear infection	4 (0.7)	2 (0.6)	1 (0.7)
Pharyngitis	4 (0.7)	3 (0.8)	1 (0.7)
Influenza	4 (0.7)	4 (1.1)	–
Primary hypothyroidism	3 (0.5)	1 (0.3)	2 (1.5)
Adrenal insufficiency	3 (0.5)	–	3 (2.2)
Abdominal pain	3 (0.5)	1 (0.3)	1 (0.7)
Diarrhea	3 (0.5)	1 (0.3)	1 (0.7)
Pyrexia	3 (0.5)	2 (0.6)	1 (0.7)
Bronchitis	3 (0.5)	1 (0.3)	2 (1.5)
Urinary tract infection	3 (0.5)	1 (0.3)	2 (1.5)
Tonsillitis	3 (0.5)	2 (0.6)	1 (0.7)
Blood thyroid-stimulating hormone increased	3 (0.5)	1 (0.3)	–
Hypoglycemia	3 (0.5)	1 (0.3)	2 (1.5)
Urticaria	3 (0.5)	1 (0.3)	2 (1.5)
Adverse events considered GH-related	14 (2.3)	11 (3.1)	2 (1.5)
Adverse events not considered GH-related/unknown	116 (19.0)	54 (15.1)	40 (29.6)

^a^Calculated for the total modified safety population (all 700 GH-treated patients, the 416 patients with idiopathic GHD and the 148 patients with organic GHD)

^b^Treatment-emergent adverse events occurring in ≥0.5 % of patients overall. Individual terms are MedDRA preferred terms and are as coded by investigators at each site. Multiple terms may therefore have been selected from when classifying an event

^c^Site did not provide distinction between primary, secondary and tertiary events

Among 612 GH-treated patients with at least one post-baseline study visit, 130 (21.2 %) had at least one treatment-emergent adverse event reported (Table 4). Of these, 14 (2.3 %) patients experienced events considered by the investigators to be GH-related. The GH-related events occurred mainly in patients with a diagnosis of idiopathic GHD. No GH-related treatment-emergent events were reported in the 42 patients with Turner syndrome and at least one follow-up visit. The most frequent specific treatment-emergent adverse event considered related to GH therapy was headache (in 3 patients [0.5 %]), followed by arthralgia (2 patients [0.3 %]) and impaired fasting glucose (2 patients [0.3 %]). In addition, six patients (1.0 %) experienced metabolism and nutritional disorders, such as obesity and alterations in glucose levels, considered related to GH treatment. Specific neoplastic events were reported for 3 patients; these were two girls with Turner syndrome who experienced melanocytic nevi, considered unrelated to GH treatment, and the boy with craniopharyngioma recurrence.

## Discussion

The effects of GH therapy at 1–4 years of treatment and at final height were studied in a country-specific cohort of pediatric patients treated for short stature. The data examined were for children in Italy entered in the GeNeSIS surveillance program. At study entry, mean height SDS was below −2, overall and for each of the diagnostic groups, and height SDS was below the target defined from parental heights. GH therapy reduced the height deficit at the assessments within the first few years of treatment and at final height for patients in individual diagnostic categories, with no adverse event concerns observed.

The primary diagnosis associated with short stature was GHD for the majority of the children (85.5 %), with the next most common being SHOX deficiency including Turner syndrome (7.6 %). The percentage of patients with GHD in Italy was higher than in the global GeNeSIS population (63.8 %), which used the same definition of GHD [23], although within the GHD diagnosis the proportions with idiopathic (73.5 %) and organic (26.1 %) causes were comparable to those in the global population (77.7 and 22.0 %, respectively). In contrast, the proportion of patients with SHOX deficiency in Italy was lower than in the global study population (11.7 %). It should be noted that the global GeNeSIS population included 13.0 % of patients with idiopathic short stature, which is not an approved indication for GH therapy in Europe and would, therefore, have influenced the proportions of patients in the other diagnosis groups.

The patients with organic GHD were younger at start of GH treatment than those with idiopathic GHD. Mean HV, height SDS and gain in height SDS were similar for the two GHD groups over the first year through to 4 years of treatment. However, for patients with available data at final height, duration of GH treatment was longer and mean gain in height SDS was greater for the organic GHD group than the idiopathic GHD group, suggesting that age at treatment start and duration of treatment may have influenced final height. The present results are in agreement with other reports that height gain is affected by factors including age at start of GH treatment and baseline target height deficit [26–29].

At final height, both GHD diagnostic groups had height SDS close to target height, even though the baseline target height deficit was greater for the organic group than the idiopathic GHD group. A final height SDS greater than −2 was achieved in 94 % of patients with organic GHD and 88 % of patients with idiopathic GHD. Interestingly, for patients who reached final height, the GH dose per body weight was lower for the organic GHD group than for the idiopathic GHD group. The greater response with lower dose in the organic GHD group relative to the idiopathic GHD group was contrary to reports that response is greater with a higher dose [3, 28, 29], although others have also found that GH dose was not a predictor of adult height [27]. However, the difference in dose may reflect the more severe deficiency seen in the organic GHD group compared with the idiopathic GHD patients, and the corresponding better response to GH replacement.

In the Turner syndrome group, HV increased from 3.50 cm/year before GH start to 7.43 cm/year in the first treatment year, similar to previously published results [30]. A mean gain in height SDS was seen, but this was less than for either GHD group at all time points evaluated. However, the mean age at which GH was initiated was relatively high and similar to that in the idiopathic GHD group. Early intervention for short stature, before the age of 4 years, has been reported to provide a better response [4, 31]. A slightly higher mean GH dose of 0.05 mg/kg/day (=0.35 mg/kg/week) has been used in some other studies of Turner syndrome [4, 5, 31]; however, lower doses of 0.23–0.26 mg/kg/week have also been utilized [30]. Administered dose varies widely, and the aim is to provide an effective dose that is well tolerated and without increased risk of adverse events [6, 13]. Final height SDS remained below target height, with a mean deficit of −1.49 SDS, although a final height SDS above −2 was achieved in 62 % of patients in the Turner syndrome group, who were treated for a mean of 5.1 years. These figures were very similar to a previous report where 62 % of Turner syndrome patients treated for at least 3 years had a final height SDS >−2, compared with 40 % when the treatment duration was only 1 year [32].

Although the number of patients in the Italian GeNeSIS cohort was not as large as for multinational databases, adverse events, overall and for specific diagnostic categories, as documented by the investigators, did not present any new concerns and were consistent with the known profile for pediatric GH treatment [13, 14]. This finding is important, because a recent Delphi survey showed that Italian pediatric and adult endocrinologists follow current guidelines and continue or reinstitute GH treatment in patients with confirmed GHD in the transitional period from adolescence to early adulthood [33], potentially prolonging the duration of GH administration. In the Turner syndrome group, no serious adverse events were identified and there were no GH-related treatment-emergent events. However, there were two reports of melanocytic nevi, which were considered by the investigators to be unrelated to GH therapy; melanocytic nevi are a common dermatological finding genetically associated with Turner syndrome irrespective of GH treatment [15, 34, 35]. Other studies have also reported very few adverse events in GH-treated patients with Turner syndrome [4, 30, 31], although headache, intracranial hypertension and slipped capital femoral epiphysis occurred more frequently in GH-treated children with Turner syndrome than in those with idiopathic GHD [16]. One death was documented, which was due to respiratory failure and unrelated to GH treatment. There was also one discontinuation in relation to impaired glucose tolerance, which is known to be associated with GH therapy [15, 17, 18].

Because of the observational nature of GeNeSIS, new patients were added to the database over time, which meant that the number of patients with long-term data was less than for short-term therapy. Thus, only major diagnostic categories could be assessed at 4 years and at final height, which was a limitation. All information entered in the database was at the discretion of the investigators; thus, not all information for each patient was available, and information for untreated control patients was insufficient for comparison. We also did not have sufficient data concerning birth details (e.g., birth weight and length), genetic testing results and use of hormone replacement therapies that may have affected final height of the children in our study. Nevertheless, we are not aware of any other reports of auxology of Italian GH-treated children and believe that the results can be considered as representative of GH therapy in standard endocrine practice in Italy.

In conclusion, data from the Italian cohort of GeNeSIS provided auxological and safety results for GH therapy that were consistent with those from other clinical trials and international surveillance databases. Height gains were observed in the first year of GH treatment through to attainment of final height. Mean height gain was more pronounced in patients with idiopathic or organic GHD than in girls with Turner syndrome, and patients with GHD achieved a mean final height that was very similar to target height. Patients with organic GHD started GH treatment at a younger age than patients with other diagnoses and were treated for longer, resulting in the largest gains in final height.
